# Gait Analysis in Walking and Trotting Dairy Cows on Different Flooring Types with Novel Mobile Pressure Sensors and Inertial Sensors

**DOI:** 10.3390/ani12182457

**Published:** 2022-09-16

**Authors:** Daniela Fischer, Luise I. G. Friebel, Sarah Grund, William Winter, Franziska C. Wagner, Christoph K. W. Mülling

**Affiliations:** 1 Institute of Veterinary Anatomy, Histology and Embryology, Faculty of Veterinary Medicine, Leipzig University, 04103 Leipzig, Germany; 2 Faculty of Computer Science and Media, HTWK Leipzig University of Applied Sciences, 04251 Leipzig, Germany

**Keywords:** dairy cow, claw health, rubber mats, concrete flooring, sensor technology, center of pressure, inertial measurement unit, accelerometer

## Abstract

**Simple Summary:**

To study effects of housing conditions on the claw health of dairy cows, objective gait analysis methods can be useful. In this study, a novel mobile pressure sensor system, attached under the claws of the hind limbs of dairy cows, was used for the first time. Additionally, inertial measurement units (IMUs) in combination with a newly developed automatic step detection algorithm were used. Gait analysis was performed in ten dairy cows, walking and trotting on concrete and rubber mats. The results showed the applicability of the objective gait analysis methods in dairy cows. Analysis of pressure under the claws revealed a significantly higher load in cows moving on concrete compared to rubber mats. The development of objective methods should be used to gain knowledge about factors that impair claw health. Sensor-based research should be applied to improve animal welfare by evaluating the housing environment objectively and better adapt it to the cows’ needs.

**Abstract:**

Mechanical overburdening is a major risk factor that provokes non-infectious claw diseases. Moreover, lameness-causing lesions often remain undetected and untreated. Therefore, prevention of claw tissue overburdening is of interest, especially by analyzing harmful effects within dairy cows’ housing environment. However, objective “on-cow” methods for bovine gait analysis are underdeveloped. The purpose of the study was to apply an innovative mobile pressure sensor system attached at the claws to perform pedobarometric gait analysis. A further goal was the supplementation with accelerative data, generated simultaneously by use of two inertial measurement units (IMUs), attached at metatarsal level. IMU data were analyzed with an automatic step detection algorithm. Gait analysis was performed in ten dairy cows, walking and trotting on concrete flooring and rubber mats. In addition to the basic applicability of the sensor systems and with the aid of the automatic step detection algorithm for gait analysis in cows, we were able to determine the impact of the gait and flooring type on kinematic and kinetic parameters. For pressure sensor output, concrete was associated with significantly (*p* < 0.001) higher maximum and average pressure values and a significantly smaller contact area, compared to rubber mats. In contrast to walking, trotting led to a significantly higher force, especially under the medial claw. Further, IMU-derived parameters were significantly influenced by the gait. The described sensor systems are useful tools for detailed gait analysis in dairy cows. They allow the investigation of factors which may affect claw health negatively.

## 1. Introduction

Lameness and claw lesions are some of the most concerning problems in dairy cow farming [1]. For decades, the resulting welfare and economic issues have been known and investigated, but herd prevalence is still at a high level [2]. The etiology of typical non-infectious claw diseases such as sole ulcers is multifactorial. In addition to anatomical predispositions [3], claw conformation [4], time around parturition, metabolic factors, the surface of walkways and locomotion influence the development of pressure-related claw horn lesions [5,6]. Many efforts have been made to develop automatic lameness detection systems [7,8]. However, the detection of lameness often does not lead to treatment, which could be due to an underestimation of the problem, or financial and management reasons [9]. This highlights the importance of a better understanding of mechanisms that damage the claw tissue of modern housed dairy cows and improving preventative measures. The overall objective is the prevention of subclinical lameness.

Sensor technology provides the possibility to analyze the impact of flooring surfaces on the claw during walking [10], objective assessment of behavior associated with limb pathologies [11] and knowledge about detailed bovine locomotion characteristics [12] and may be useful to assess animal welfare [13]. Up to now, most studies analyzing the weight, force or pressure distribution under the bovine claw were conducted with stationary measurement platforms, so called “off-cow sensors” [14]. These systems allow a more accurate examination of the load in comparison to wearable sole-shaped sensors [15]. However, due to technical, financial or spatial requirements, the applicability of measurement platforms is limited [16]. 

Inertial sensors such as accelerometers are used in behavioral research and their application is widespread in livestock farming [13]. They are suitable for high-frequency gait analysis in dairy cows [17,18,19] and allow a low-cost on-cow approach [20].

Since there are no commercial on-cow devices to study the claw–floor interaction under the bovine claw, we used a novel mobile pressure sensor system to investigate kinetic parameters during locomotion. To verify temporal parameters and analyze the kinematics of gait, inertial measurement units (IMUs) were used additionally. 

The purpose of this research was the preliminary application of a newly developed sensor system. We hypothesized that the novel pressure sensor system and the IMU, together with a newly developed pattern recognition algorithm, are suitable for gait analysis in dairy cows. With the use of both sensor systems, we analyzed the influences of gait (walk and trot) and flooring surfaces (concrete and rubber mats) on kinetic and kinematic locomotion parameters.

Besides this scientific goal, we tested the general applicability and durability of the novel pressure sensors and the attachment under field conditions with the cows walking and trotting across hard flooring. 

## 2. Materials and Methods

### 2.1. Pressure Sensor System

**Figure 1:**
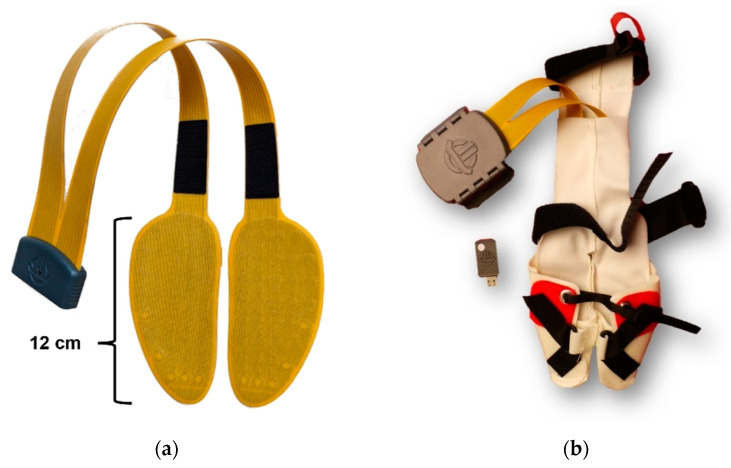
Sensor systems used for gait analysis in ten dairy cows: (**a**) paired claw-shaped pressure sensor soles; (**b**) textile claw shoe for attachment of the sensors.

A novel mobile pressure sensor system (UngulaPress©, Thorsis Technologies GmbH, Magdeburg, Germany) was used for the first time for gait analysis in dairy cows. It consists of flexible, paired, claw-shaped sensor soles (Figure 1a) and wireless modules for data transfer, which enabled a WLAN connection via a USB dongle, as well as the analysis software VisuPress© Ungula© (version 1.1.13 to 1.1.30, Thorsis Technologies GmbH, Magdeburg, Germany). Measurement area of one claw-shaped sensor sole was 61 cm^2^ and included 35 piezoresistive single sensor cells. Measurement was performed with a sampling rate of 30 Hz. The sensor cells as well as the conductor tracks were embedded in a waterproof multilayer robust textile cover. Sensor soles were inserted into an innovative textile claw shoe to achieve fixation under the bovine claw (Figure 1b). The equipment was attached in a trimming chute, with the respective limb raised. Claw shoes were fixed and adjusted with Velcro straps to allow a precise fitting. 

The sensors were magnetically held and connected to the module, which had an average battery life of 4 h. The module was manually activated and protected by a solid plastic box, which could be attached at the cow’s metatarsus with a Velcro strap. For each cow, new sensor soles were used. The analysis software allowed monitoring pressures in live mode as well as storage, analysis and export of raw data after measurement.

### 2.2. Inertial Measurement Units (IMUs)

**Figure 2:**
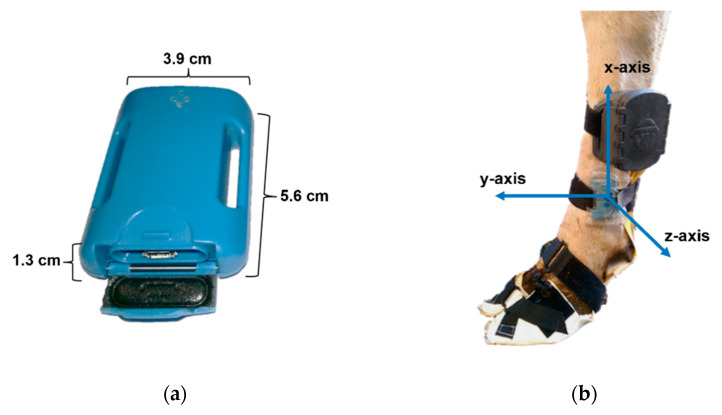
(**a**) Inertial measurement unit (IMU) dimensions; (**b**) orientation of an IMU (blue arrows) attached to the metatarsus above the claw shoe of the left hind limb.

Two IMUs (HAM-IMU, Gulf Coast Data Concepts, LLC, Waveland, MS, USA; Figure 2a) were used to validate the temporal data of the novel pressure sensor system and to increase the validity of gait analysis with kinematic data. Each IMU weighed 25 g and had a default sampling frequency of approximately 226–228 Hz. The integrated triaxial accelerometer operated with a default range of ± 16 g, the gyroscope was set to measure the angular rate of change up to 1000 °/s. Magnetometer output and quaternions were not used for analysis. After activation, both IMUs were knocked together in front of an action camera (setting: 120 fps, active mode, linear; HERO8 Black, GoPro^®^, Inc., San Mateo, CA, USA) to allow synchronization with the video recordings [19]. Video analysis was performed using the open-source research software BORIS [21]. An IMU was attached above the claw shoe on each hind limb, proximal to the fetlock joint, on the lateral side of the metatarsus (Figure 2b). The x-axis was orientated vertically, y-axis horizontally and z-axis in transversal direction. 

### 2.3. Animals

Ten adult lactating Holstein–Friesian dairy cows from the Oberholz Farm for Teaching and Research at the Faculty of Veterinary Medicine, Leipzig University were used for gait analysis. The experiments were performed with permission of the local authority (ethical approval, TVV 24/19, Landesdirektion Sachsen, Chemnitz, Germany) as a preliminary test of the project “KlauSens”. The herd was housed in a free stall barn with straw bedded cubicles and mastic asphalt walkways, and lactating cows were milked twice a day. Cows in the third trimester of pregnancy and cows ≤ 4 weeks post-partum were not included in this study. Only clinically sound and lactating dairy cows were selected. The cows had an average bodyweight of 713.2 kg ± 80.9 kg and were between 2.3 and 5.2 years old, with an average of 3.6 ± 1.0 years. The body condition score was determined with a 5-point scale [22], scored by an experienced veterinarian in consultation with two other veterinarians. The median value of the body condition score was 3.0 ± 0.5. The cows in this study had a locomotion score ≤ 2, according to Sprecher et al. [23]. Claws of the hind limbs were examined for pathologies in the trimming chute. Only cows with sound claws and parallel position of the hind limbs were included in the study, to reduce the risk of unnecessary pain due to subclinical lameness, and for reasons of a better fit of the textile claw shoe. Based on this inclusion criteria, the most cooperative cows were chosen, which were assumed to experience as little stress as possible, e.g., while standing in the trimming chute. The last routine claw trimming of the herd was performed five months before the trials. Claw trimming was not performed directly before the measurements, because the claws of the selected cows were in good condition, and it was not intended to study recently trimmed claws.

### 2.4. Experimental Setup

**Figure 3:**
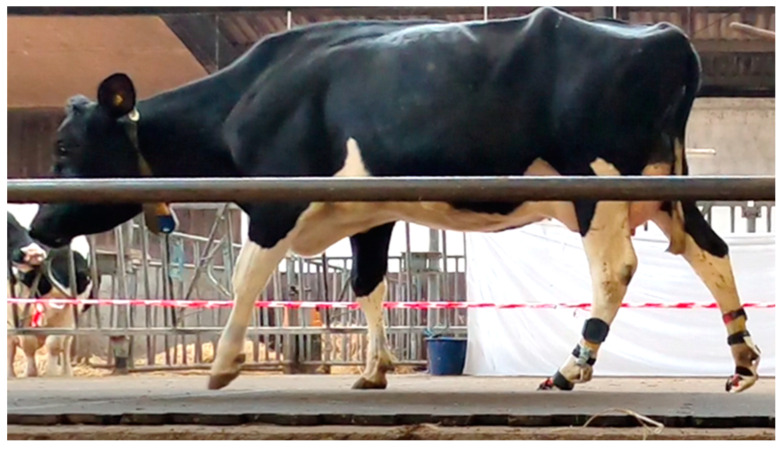
Cow walking across the walkway with rubber mats during the trial.

Measurement systems were attached to the hind limbs in a trimming chute. Animals were treated calmly to avoid unnecessary stress. After the attachment of the equipment, cows were released calmly from the trimming chute, and they were given a few rounds to get used to the claw shoes until they showed a good acceptance of the equipment (no shaking of the hind limbs or sniffing at the shoes). The experiments were performed on two walkways, build up at the feeding table. Each walkway was 15 m long and 1.5 m wide. One had a twenty-year-old solid concrete surface without abrasive grit on the surface or protective coating. The second walkway was covered with rubber mats (KURA P, Gummiwerk KRAIBURG Elastik GmbH & Co. KG, Tittmoning, Germany). During measurements, the walkways were kept dry and clean, to prevent the surface from becoming slippery. Both walkways were connected to each other at the end to allow the cows to walk and trot several rounds without turning around (Figure 3). One or two persons walked behind and/or in front of the cow, to obtain a steady gait, and to prevent more active cows from changing into a faster gait or calmer cows from stopping or slowing down. Depending on the cows’ temperament and in the case of unsteady gait, the animals had to walk the rounds repeatedly. To obtain sufficient data, animals had to pass each walkway four times in each gait. Walking and trotting were documented by three veterinarians present during the measurement. Walking speed was not determined. Cows were not specifically trained to walk with the equipment. After measurement, cows were released into their herd, where the equipment could be removed easily, e.g., while the cow was standing in the cubicle or at the feeding lot.

### 2.5. Pressure Data Analysis

Obtained pressure data of the entire measurement period of each cow were screened to select subsequences, which contained an average of 5.2 consecutive steps (range: 3–8 steps). Using markers, set in the live mode during the measurement in the VisuPress© Ungula© software, as well as video recordings, the sequences were assigned according to the respective combination of gait (W = walk/T = trot) and flooring type (C = concrete/R = rubber mats). The manual selection of step sequences was performed to exclude interruptions such as standing or turns at the end of the walkway, so only steady gait sequences were used for analysis. Sections with unclear gait cycles or transitions between walk and trot were not used for data analysis.

The raw data were exported, and the parameters contact area (A), total pressure and maximum pressure (pMAX) of the stance phases were analyzed using a custom-written script in the software MATLAB (version R2020b, The MathWorks, Inc., Natic, MS, USA). Further, the parameters average pressure (pAVG) and force (F) were calculated for each claw (LC = lateral claw, MC = medial claw) and each limb (total), from A and the total pressure. The balance of force (FB) was analyzed relatively within each limb.

The center or pressure (COP) was calculated as the center of weighted points in an area. Two types of center of pressure were determined based on the known length of the sensor sole: the COP of each hind limb (COP_HL) and the COP of each single claw (COP_CL). For every trial, the path of the COP_HL was examined separately for both hind limbs. The shape of the COP path during stance phase was analyzed, also regarding mirrored patterns between the two hind limbs, and the craniocaudal extent of the COP_CL was measured. The patterns used for COP evaluation were scaled to the claws’ sizes by using photo documentation.

### 2.6. Inertial Measurement Unit Data Analysis

The data were analyzed with a custom-written MATLAB script. Conversion of raw data to g-values was performed according to the manufacturer’s manual [24]. Since the sampling rate variated slightly during measurement, data were resampled to 200 Hz. 

A pattern recognition algorithm was written to segment step cycles based on peak detection. For this purpose, the steps were first segmented based on the acceleration of the y-axis. The calculated segments were transferred to the total acceleration (acc), which was calculated as the vector sum of the acceleration in *x*-, *y*-, *z*-axes [18,25,26,27], and the maximum peaks of the acceleration were defined as touch down. In the z-axis of the gyroscopic sensor, a point was found that represents the toe’s take off (terminal ground contact). This point was a saddle point between the minimum and maximum of a step. The gait parameters could then be calculated using these points. Derived from acc and the terminal ground contact, the parameters listed in Table 1 were used for further analysis.

Gait sequences were labeled according to gait and flooring type (W-C/W-R/T-C/T-R) by the aid of video recordings. Gait patterns were derived from pressure data and the markers set during measurement in the VisuPress© Ungula© software.

### 2.7. Statistical Analysis

Statistical analysis of pressure and IMU parameters was performed using nlme and stats packages in R (version 4.0.2 [28]) with RStudio (version 1.3.1093, RStudio Team (2020), PBC, Boston, MA, USA). For model comparison, the models “lm_mod1”, “gls_mod1”, “lme_mod1_randint_only” and “lme_mod1” were tested. The best-fitting model for each parameter was selected with an ANOVA. 

For pressure data, the factors “cow” and “limb side” were considered with a random slope for all models, because of the assumption of interindividual random effects. Due to a left-steep distribution, data were transformed by log2. After model selection, the influences of the factors “claw” (LC/MC), “gait” (W/T) and “floor” (C/R) towards the parameters A, pMAX, pAVG, F were examined with a linear mixed-effects model. The interactions “claw*limb side” and “claw*gait” were considered for the parameter F. For parameter A, only the interaction “claw*limb side” was considered. The models for pressure data analysis were adjusted by the maximum restricted likelihood method. 

For IMU parameters, the influences of the factors “gait”, “floor” and “side” were tested. After model comparison, the selected linear mixed-effects model was adjusted by the maximum likelihood method. A random slope for “cow” was considered.

The length extent of the COP_CL relative to total claw length is a dependent parameter (claws of the same limb and limbs of the same cow). Further, the data of all animals did not show a normal distribution. Thus, the Wilcoxon signed rank test was used for the data analysis. An alpha of *p* < 0.05 was defined to determine statistical significance.

## 3. Results

### 3.1. Pressure Data Analysis

The contact area A was influenced by the factors flooring (*p* < 0.001), claw (*p* < 0.001) and gait (*p* = 0.008). The lateral claws were associated with the biggest contact area, which measured on average 46.4 cm^2^ at “trot on rubber mats” (T-R). The smallest value for A under LC was 36.6 cm^2^ and was found at “walk on concrete” (W-C).

The parameter pMAX was found to be influenced by the factors gait (*p* < 0.001), flooring (*p* < 0.001) and claw (*p* < 0.001). The highest values were found under the medial claws (MC) at “trot on concrete” (T-C) with an average of 253.3 N/cm^2^. Compared to “walk on rubber mats” (W-R), pMAX measured for W-C was approximately 75% higher under the LC. 

**Figure 4:**
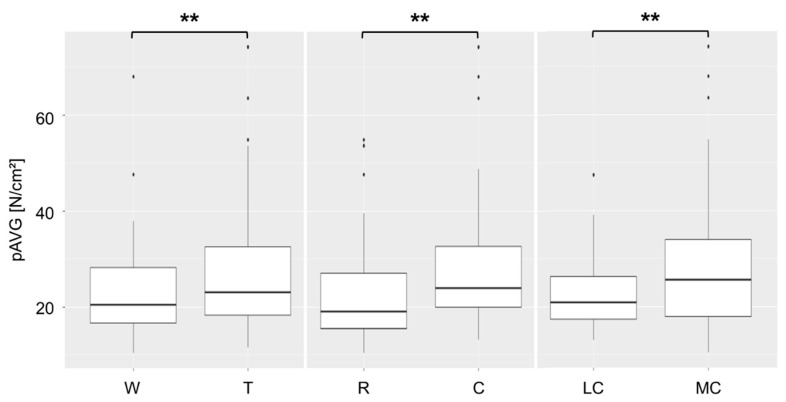
Influence of the factors gait (W = walk, T = trot), flooring (R = rubber mats, C = concrete) and claw (LC = lateral claw, MC = medial claw) on the average pressure (pAVG); the mean values of subsequences with 3 to 8 consecutive steps from ten dairy cows are plotted. ** indicates a significant influence of the different factors, *p*-value < 0.001.

The factors trot, concrete and the medial claw led to a significant increase in pAVG (Figure 4). The highest average pressure values were found with 35.4 N/cm^2^ at T-C under the MC. The lowest pAVG was found for LC at W-R with a mean of 18.9 N/cm^2^. 

**Figure 5:**
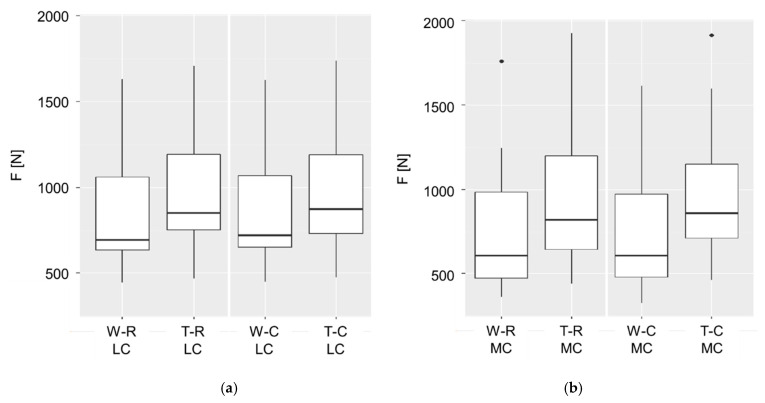
Influence of the interacting factors gait (W = walk, T = trot) and flooring (R = rubber mats, C = concrete) on the force F, shown for (**a**) lateral claws (LC) and (**b**) medial claws (MC); shown are the mean values of subsequences with 3 to 8 consecutive steps from ten dairy cows (modified from Fischer *submitted* [29]).

F was significantly affected by gait (*p* < 0.001), interaction of MC*T (*p* < 0.001), interaction of MC*right side (*p* = 0.008) and by the side (*p* = 0.018). The lateral claw was generally more loaded, except at trot, as it is shown in Figure 5. The effect of gait was stronger on the medial claw. The highest force under the limbs was found at T-C with an average of 1959.2 N.

The force balance was always at the load of the lateral claw. On average, the LC was loaded with 50.8 % (T-C) to 54.4 % (W-C) of the total force within each limb. The mean values for each parameter for walk can be found in Table 2, the values for trot in Table 3.

Three different shaped COP_HL patterns were found (L-form, S-form, square bracket and their mirrored forms). Mirrored COP_HL patterns between both hind limbs were found in six out of ten cows at walk and in seven out of ten cows at trot. On rubber mats, six out of ten cows showed the same COP_HL pattern at walk and trot. On concrete, three out of ten cows showed the same pattern in both gaits. At walk, similar COP_HL patterns on both surfaces were found in seven out of ten cows. At trot, five out of ten cows showed the same pattern.

**Figure 6:**
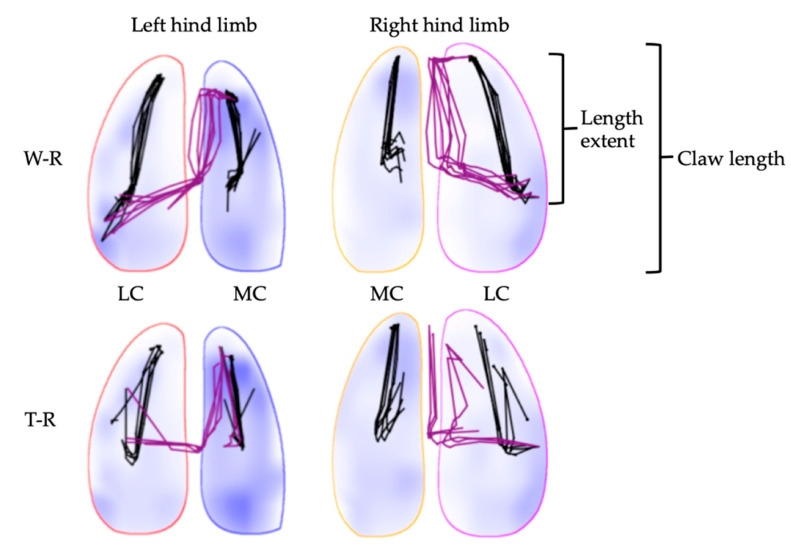
COP_HL (purple) and COP_CL (black) data of one cow are shown for walk (W-R) and trot (T-R) on rubber mats. In this case, the COP_HL pattern is mirrored between both hind limbs for both gaits (L, mirrored L).

At walk, the length extent of the COP_CL relative to the total claw length was similar on concrete (LC: 58.6 ± 10.8%, MC: 55.8 ± 9.8%) and on rubber mats (LC: 59.3 ± 12.8%, MC: 54.0 ± 11.3%). Similar results were found at trot on concrete (LC: 47.1 ± 8.6, MC: 44.7 ± 8.1%) and rubber mats (LC: 44.6 ± 8.2%, MC: 42.2 ± 6.9%). In contrast, significantly longer COP_CL extents were detected at walk compared to trot on both concrete (LC: 12.9 ± 3.6%, MC: 10.6 ± 6.0%) and rubber mats (LC: 14.8 ± 7.0%, MC: 11.7 ± 6.5%). Hence, the length extent in caudal direction shortened. An example for both COP data is shown in Figure 6.

### 3.2. Inertial Measurement Unit Data Analysis

**Figure 7:**
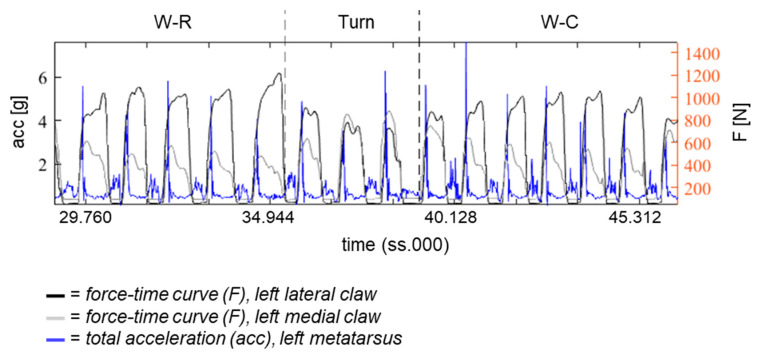
Combined visualization of force (F) and IMU data (acc). Exemplary gait pattern of the left hind limb of one cow is shown. Labels (W-R = walk on rubber mats, turn, W-C = walk on concrete) were transferred from the markings during measurement.

Events such as initial and terminal ground contact of step cycles were automatically identified by finding the peaks in the curve of acc [18] and a saddle point in the curve of gyr_z. These events were verified using video analysis. An example of a combined gait pattern of force and IMU data is shown in Figure 7. By screening data of the measurements, it could be found that peaks at initial ground contact did not exaggerate the 16 g measurement limit of the triaxial accelerometer.

Due to technical issues (unexpected failure of the lithium-ion battery under temperatures), not all data sets could be analyzed for all testing conditions (Cow*Side*Floor*Gait). Therefore, only 60 observations were included in the analysis of IMU-derived parameters, 20 observations were not exploitable.

**Figure 8:**
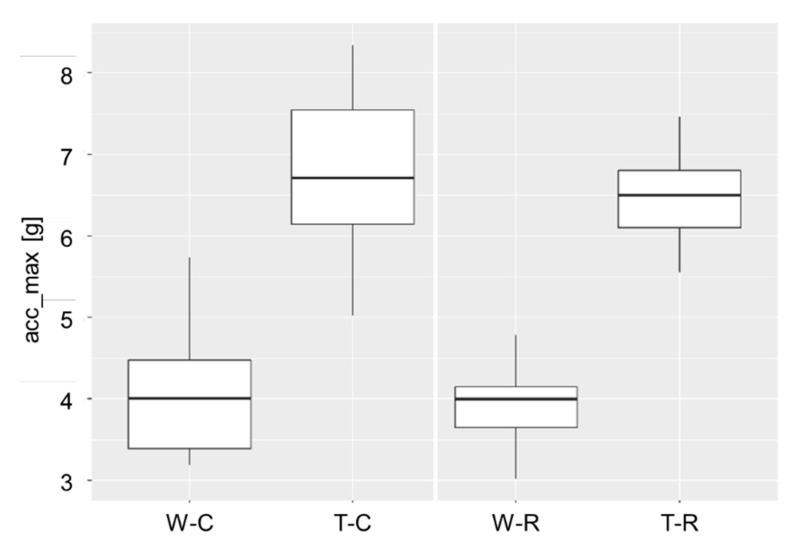
Influence of the factors gait (W = walk, T = trot) and flooring (R = rubber mats, C = concrete) on the peak acceleration acc_max at initial ground contact; mean values of 60 observations (mean values of automatically detected steps of the labeled gait sequences) from ten dairy cows are plotted.

Statistical analysis revealed a significant influence (*p* < 0.001) of the factor gait on all parameters. The peak of the total acceleration (acc_max) reached its highest average value at T-C, with 6.7 g, see Figure 8. The lowest value for acc_max was found at W-R with an average of 3.9 g. 

Furthermore, the other parameters were not influenced by the factors floor (C or R) or side (HL or HR). Except for the parameter gyr_z, the left hind limb was associated with higher values (*p* = 0.003), compared to the right hind limb. 

For the temporal parameter dur_step, mean values of 1.26 s (W-C and C-R) and 0.74–0.75 s (T-C and T-R) were found at walk and trot, respectively. The relative duration of the stance phase was 69.7% at W-C and 69.9% at W-R. At trot, lower values for rel_stan were found in comparison to walk (58.2% for T-C and 58.9% for T-R). Mean values of 60 observations from ten dairy cows are shown in Table 4 for walk and in Table 5 for trot.

## 4. Discussion

The novel pressure sensor system was used successfully for gait analysis in cows on the hind limbs on hard and soft flooring. Although all cows visibly became used to the claw shoes after a few rounds, wearing the shoes themselves may have biased locomotion. Additionally, it must be noted that locomotion is influenced by the cows’ motivation to walk [30]. By training the cows to walk steady without guidance, the output of utilizable data could have been improved as well as by using longer walkways. It could not be excluded that the equipment became wet or polluted, whereas the walkways remained clean for measurement to prevent slipperiness. The hardware and the claw shoes resisted external influences such as moisture and shear forces under the bovine claw during the measurements and until take off inside the barn. The measurement frequency as well as the computational resolution of 0.57 sensor cells per cm^2^ should be increased for further measurements, to allow a meaningful clinical interpretation of pressure distribution, e.g., within different step phases [31] and claw regions [32]. For comparison, the sensor resolution of mobile sensor soles for human gait analysis ranged from 0.5 to 4 sensor cells per cm^2^ [33]. 

Analysis of different gaits and floorings demonstrated the applicability of this system to evaluate the influence of different flooring conditions on locomotion behavior. To the authors’ knowledge, this is the first study to measure dynamic pedobarometric parameters in walking and trotting cows on concrete and rubber mats. Nevertheless, it must be considered that due to the small size of the study and the special conditions of a teaching and research farm, the results should not be considered representative for conventional farms. Due to technical limitations, it was not possible to record pressure data for more than two limbs. The examination of the front limbs or ipsilateral limbs would have been technically possible as well, but the hind limbs were focused on due to expected better comparability with the cited studies. Although the present study included only the hind limbs, we consider it useful to perform pressure analysis under the forelimbs as well, or even all four limbs, if relevant for the scientific question. 

When comparing the data with the results of other studies, it should be noted that there are methodological differences between wearable sensors and pressure plates: stationary pressure plates only record vertical load, whereas wearable sensor soles are flexed and record loads that are not necessarily vertical to the ground, e.g., during foot-loading or take off [15,34]. In addition, the experimental setup differs depending on the type and positioning of sensors, which should be considered when interpreting absolute values. Sole-like pressure sensors allow flexible application and analysis of many consecutive steps, while pressure plates often require repeated measurement passes [31]. 

In accordance with Oehme et al. [10], who also used mobile on-cow sensors but a different type, we found increased pAVG and pMAX values and decreased contact area under the bovine claw for concrete flooring, compared to rubber mats in walking cows. Pressure parameters are influenced by rough floorings such as concrete, which is considered to harm the sole horn due to high peak pressures, as found in an ex vivo study on bovine limbs [35]. Telezhenko et al. [36] provided an ex vivo approach for measuring the pressure inside the claw and they found significantly higher pressures in the lateral sole zone on concrete, in contrast to pasture surface. The results from Oehme et al. and Oehme [10,37] show the increase in mean and maximum pressures under dynamic conditions compared to static measurements. This highlights the importance of dynamic gait analysis with respect to claw–floor interaction, especially in the immediate environment of the cows. It seems plausible that the system presented here could also contribute to a better understanding of the mechanical-related etiology of claw horn disruption. 

The analysis of force exerted on the sensor soles showed significant differences between the gaits walk and trot. This leads to the assumption that faster movement as well as sudden turns or sliding may result in harmful load. Excessive loads or impacts during running, jumping or sliding may be more harmful than steady walking. Those, possible severely harmful, events may be important for further developments of rubber mats and should therefore be considered when studying gait. Therefore, unassisted gait analysis while moving freely inside the barn with interactions with other cows may be useful. The force distribution between the medial and lateral claw was more balanced at trot, compared to walk. On concrete, the medial claw carried more relative load than on rubber mats (on average + 0.4% at walk and +1.0% at trot). This effect could be due to the interaction found between medial claw and trot for the parameter F. Further analysis of the localization of pMAX showed that, especially at trot, high forces were found in the toe area of the MC. This supports the evidence that the medial claw contributes to weight bearing in the rear part of the stance phase [31,38] and leads to the assumption that the toe tip of the medial claw is the last point of ground contact. In contrast to van der Tol et al. [31], who performed measurement with a stationary pressure and force platform, we used flexible pressure sensor soles. This type of sensor could have led to these high force and pressure values, especially during push off [33].

Regarding the measurement of the COP, Nauwelaerts et al. [39] found that the ideal COP path does not exist. In our study, more than half of the cows showed a mirrored COP_HL pattern between both hind limbs, which is consistent with Nauwelaerts et al. [39]. At walk, the flooring surface does not seem to have a significant influence on the COP_HL pattern. On rubber mats, six out of ten cows showed the same COP_HL pattern at both walk and trot. On concrete flooring, however, this was the case for three cows. It seems plausible that a hard surface leads to greater variability and thus possibly to different pressure distributions. An increased risk for the development of mechanically traumatic claw lesions would be conceivable. The COP pattern is the result of multiple optimization processes occurring simultaneously [39]. Additionally, variations in foot structure are also reflected in different patterns of dynamic load distribution under the foot [40].

To the authors’ knowledge, no other studies considered the COP of the individual claws. The flooring surface had no influence on the length extent of the COP_CL relative to the total length of the claw. In contrast, an influence of gait was shown: trotting resulted in a significant reduction in length extension compared to walking. Due to the higher speed, the claws are possibly placed more planar and thus the propulsion phase is shortened. This reduces the extent in the caudal direction. Initial contact during footing is made with the lateral claws. The medial claws are increasingly loaded from the mid-stance phase, which may explain the shorter length extent of the COP_CL. Further analysis of COP path influencing effects could lead to better understanding of footing biomechanics. COP analysis could be a useful tool to study claw trimming effects, or, for example, the effects of different flooring systems on the stature of rearing heifers.

Combined gait analysis with pressure sensors and IMU can be carried out with limitations. By using IMU as a reference system for temporal data, an error in the timing function of one pressure sensor module was detected visually. Due to the inconsistency of this technical issue, it was not possible to analyze all IMU and pressure sensor data from both limbs synchronously, as exemplarily shown in Figure 7. 

In accordance with Alsaaod et al. [19], we were also able to identify the initial ground contact, which is characterized by peak acceleration. Additionally, we managed to identify the terminal ground contact automatically by using the gyroscope. This shows that using IMU may provide improved stance phase detection, compared to the use of a single accelerometer. This is consistent with the findings by Sapone et al. [41], who found better accuracy for methods based on gyroscopic data. In their study, Sapone et al. [41] compared methods of detecting stance phase using an IMU attached to the dorsal side of the equine metacarpal cannon bone. 

Statistical analysis revealed significant differences between walk and trot for all parameters, showing that automatic differentiation of the two gaits is possible. Therefore, an automatic tool for the differentiation of walk and trot step cycles could be developed further (not part of this publication). Especially for gait analysis in an unsupervised setting, an algorithm for the assignment of walk and trot sequences may replace labor intensive video analysis. 

Chapinal et al. [42] used the average of the total acceleration of determined walking passages for the front and hind limbs to compare cows walking on concrete and on rubber mats. In contrast to the findings made by Chapinal et al. [42], we did not observe distinct differences in the mean total acceleration between the different floors. However, Alsaaod et al. [43] found a significant effect of the flooring type on accelerometer-derived gait variables by comparing pasture and artificial flooring. Analogous to their findings [43], rubber mats had no significant effect on the locomotion characteristics compared to hard flooring such as concrete in our study. One possible explanation might be the anatomical structures from the claw up towards the metatarsal bone. Functioning as shock absorbing structures, they may attenuate minor effects of the flooring type, for example, towards the peak acceleration. In addition, shock absorbing effects of the thin textile layer of the claw shoe underneath the IMU cannot be excluded. However, gait analyses on pasture showed that locomotion is improved on soft flooring, compared to artificial flooring [43]. This must be noted when evaluating cows’ locomotion, as they may show lameness less clearly on softer flooring [44]. This emphasizes the importance of routine visual examinations and the urge to adapt floorings to the animals’ needs. However, attachment at the height of the metatarsus seems to be a promising position to perform gait analysis in dairy cows, compared to IMUs attached at the collar, head or neck [17]. The parameter acc_swing was hard to interpret but may be useful to evaluate side-specific, kinematic differences during the swing phase. Further development should be made to combine IMU measurements directly with piezoresistive pressure sensors for bovine gait analysis. This could enhance the significance of COP and footing patterns and lead to better understanding of swing phase mechanisms by using IMU output. Further, the evaluation of quaternions and a magnetometer would bring new insights about the locomotion aspects of the swing phase and add value to pedobarometric studies in bovines. 

The described sensor systems were appropriate for the monitored short-term application in housed dairy cows. Further studies on free-living animals on natural soils could lead to important findings on natural locomotion behavior. In general, it is conceivable that these sensor systems could also be used in wild ungulates if the devices were adjusted, for example, to record a longer period with a reduced data volume.

## 5. Conclusions

The novel mobile pressure sensor system is a useful tool for mobile gait analysis in dairy cows. The described on-cow sensors made it possible to visualize and study the difficult-to-imagine forces that act when cows walk or trot. Significant differences between different flooring types and gaits on the pressure-related parameters were found. Concrete was associated with significantly higher pressures exerted on the claws, which may harm the claw tissue physically, or lead to lesions and a more painful locomotion. Rubber mats seemed to exert less pressure on the claw or distribute the load more evenly. 

The IMUs allowed a high-resolution analysis of the swing phase and the accelerations during the initial ground contact. The pattern recognition algorithm provided an automatic step detection and thus a highly automated data evaluation. Further usage and research of IMUs in bovine gait analysis may add additional value to pressure or force sensor-based studies, especially when implemented in the same device.

Sensor systems for gait analysis in ungulates should be established to provide a wide range of applications and routine use, user-friendly and simple data analysis. Innovative sensor systems and reasonable algorithms may support the evaluation of floor surfaces inside the barn and cubicles, improve the education of claw trimmers or trimming methods and should be used to improve claw health and thus animal welfare. For livestock farmers, the mobile sensor system can be used to draw attention to risk factors that affect claw health, and to visualize the claw–floor interaction.

## Figures and Tables

**Table 1:** Definition of IMU parameters used for gait analysis.

Parameter	Unit	Definition
acc_max	g ^1^	g-peak of the total acceleration at initial ground contact (beginning of stance phase)
acc_swing	g ^1^	Total acceleration during swing phase (mean value)
dur_step	s	Duration of complete step cycle
rel_stan	%	Relative stance phase duration; calculated from stance phase duration and dur_step
gyr_z	°/s	Magnitude of curve deflection of the angular rate of change, measured for the z-axis around the time of the terminal ground contact; automatically selected and calculated from the last local maximum to the total minimum of a step cycle

^1^ g = 9.81 m/s^2^ (gravitational constant).

**Table 2:** Mean pressure data for ten dairy cows in total and differentiated between lateral claw (LC) and medial claw (MC) during walk on concrete (W-C) and rubber mats (W-R).

		W-C				W-R			
		Mean	SD (±)	Min	Max	Mean	SD (±)	Min	Max
**F (N)**	**Total**	**1590.4**	610.3	902.6	3241.9	**1589.6**	619.8	891.9	3392.6
	**LC**	**855.3**	320.0	450.7	1626.2	**847.9**	318.4	444.0	1630.9
	**MC**	**735.1**	341.4	323.7	1615.7	**741.7**	355.8	362.1	1761.7
**FB (%)**	**LC**	**54.4**	8.6	40.9	69.8	**54.0**	8.2	37.7	71.1
	**MC**	**45.6**	8.6	30.2	59.1	**46.0**	8.2	28.9	62.3
**pAVG (N/cm^2^)**	**Total**	**25.4**	8.9	15.6	52.4	**20.3**	6.9	11.9	37.4
	**LC**	**23.7**	6.9	15.2	37.1	**18.9**	5.3	13.0	28.6
	**MC**	**27.0**	12.1	13.2	68.0	**21.6**	9.3	10.4	47.6
**pMAX (N/cm^2^)**	**LC**	**152.3**	61.3	86.5	295.8	**87.1**	33.2	41.9	180.9
	**MC**	**195.8**	78.8	99.3	400.3	**114.5**	39.0	66.2	222.0
	**Total**	**64.6**	14.8	44.1	100.3	**79.2**	12.9	58.4	105.9
**A (cm^2^)**	**LC**	**36.6**	10.0	20.6	57.7	**44.4**	8.1	26.2	58.1
	**MC**	**28.0**	8.6	15.6	47.1	**34.9**	9.0	22.3	53.9

A = contact area; F = force; FB = force balance; Max = maximum value; Min = minimum value; pAVG = average pressure; pMAX = maximum pressure; SD = standard deviation of the mean; W-C = walk on concrete; W-R = walk on rubber mats.

**Table 3:** Mean pressure data for ten dairy cows in total and differentiated between lateral claw (LC) and medial claw (MC) during trot on concrete (T-C) and rubber mats (T-R).

		T-C				T-R			
		Mean	SD (±)	Min	Max	Mean	SD (±)	Min	Max
**F (N)**	**Total**	**1959.2**	669.7	1167.8	3610.6	**1913.4**	669.3	928.7	3636.1
	**LC**	**1000.7**	377.6	474.7	1738.9	**982.9**	340.4	468.5	1708.6
	**MC**	**958.4**	355.3	461.5	1915.8	**930.6**	383.5	440.1	1927.5
**FB (%)**	**LC**	**50.8**	7.6	38.6	65.0	**51.8**	6.9	34.2	61.3
	**MC**	**49.2**	7.6	35.0	61.4	**48.2**	6.9	38.7	65.8
**pAVG (N/cm^2^)**	**Total**	**30.2**	10.0	17.8	56.0	**24.0**	8.2	14.7	43.6
	**LC**	**25.0**	8.1	14.4	47.4	**20.9**	5.5	14.2	32.6
	**MC**	**35.4**	14.2	16.2	74.2	**27.1**	12.0	11.5	54.8
**pMAX (N/cm^2^)**	**LC**	**163.6**	63.6	97.3	336.0	**100.8**	31.4	53.0	155.1
	**MC**	**253.3**	97.9	126.6	480.3	**150.7**	59.5	66.0	298.9
	**Total**	**69.4**	16.6	42.2	101.1	**82.3**	12.2	61.5	105.3
**A (cm^2^)**	**LC**	**40.6**	10.4	19.2	55.5	**46.4**	7.2	27.4	56.6
	**MC**	**28.8**	8.7	14.5	46.5	**35.9**	8.2	22.8	48.7

A = contact area; F = force; FB = force balance; Max = maximum value; Min = minimum value; pAVG = average pressure; pMAX = maximum pressure; SD = standard deviation of the mean; T-C = trot on concrete; T-R = trot on rubber mats.

**Table 4:** Mean IMU data for ten dairy cows in total and differentiated between left (HL) and right hind limb (HR) during walk on concrete (W-C) and rubber mats (W-R).

		W-C				W-R			
		Mean	SD (±)	Min	Max	Mean	SD (±)	Min	Max
**acc_max** **(g)**	**Total**	**4.05**	0.68	3.18	5.74	**3.93**	0.47	3.02	4.78
	**HL**	**3.94**	0.78	3.18	5.74	**3.86**	0.46	3.27	4.78
	**HR**	**4.16**	0.54	3.22	4.86	**4.01**	0.47	3.02	4.59
**acc_swing** **(g)**	**Total**	**1.14**	0.10	0.98	1.37	**1.11**	0.07	0.95	1.20
	**HL**	**1.15**	0.11	1.00	1.37	**1.11**	0.06	1.02	1.20
	**HR**	**1.12**	0.09	0.98	1.25	**1.11**	0.08	0.95	1.18
**dur_step**	**Total**	**1.26**	0.05	1.19	1.37	**1.26**	0.09	1.15	1.47
**(s)**	**HL**	**1.25**	0.04	1.20	1.33	**1.28**	0.07	1.15	1.40
	**HR**	**1.27**	0.05	1.19	1.37	**1.25**	0.10	1.16	1.47
**rel_stan**	**Total**	**69.68**	1.46	67.89	72.85	**69.87**	1.45	68.49	73.39
**(%)**	**HL**	**69.56**	1.08	68.27	71.74	**69.41**	0.87	68.49	71.45
	**HR**	**69.79**	1.75	67.89	72.85	**70.34**	1.74	68.72	73.39

acc_max = peak total acceleration at initial ground contact, acc_swing = mean total acceleration during swing phase, dur_step = duration of complete step cycle, Max = maximum value, Min = minimum value, rel_stan = percentage of stance phase in the total step cycle, SD = standard deviation of the mean; W-C = walk on concrete; W-R = walk on rubber mats.

**Table 5:** Mean IMU data for ten dairy cows in total and differentiated between left (HL) and right hind limb (HR) during trot on concrete (T-C) and rubber mats (T-R).

		T-C				T-R			
		Mean	SD (±)	Min	Max	Mean	SD (±)	Min	Max
**acc_max** **(g)**	**Total**	**6.72**	0.92	5.02	8.34	**6.50**	0.52	5.56	7.46
	**HL**	**6.57**	0.86	5.02	8.34	**6.60**	0.54	5.56	7.46
	**HR**	**6.87**	0.96	5.48	7.92	**6.41**	0.49	5.87	7.31
**acc_swing** **(g)**	**Total**	**1.79**	0.24	1.40	2.17	**1.79**	0.19	1.46	2.10
	**HL**	**1.78**	0.22	1.45	2.15	**1.81**	0.18	1.48	2.07
	**HR**	**1.80**	0.26	1.40	2.17	**1.78**	0.21	1.46	2.10
**dur_step**	**Total**	**0.74**	0.06	0.66	0.85	**0.75**	0.05	0.69	0.84
**(s)**	**HL**	**0.75**	0.05	0.67	0.83	**0.75**	0.05	0.69	0.84
	**HR**	**0.73**	0.06	0.66	0.85	**0.76**	0.05	0.69	0.81
**rel_stan**	**Total**	**58.22**	3.20	50.64	63.81	**58.85**	2.68	54.31	63.48
**(%)**	**HL**	**58.41**	3.84	50.64	63.81	**57.63**	2.91	54.31	61.93
	**HR**	**58.03**	2.37	53.27	61.17	**60.07**	1.72	57.77	63.48

acc_max = peak total acceleration at initial ground contact, acc_swing = mean total acceleration during swing phase, dur_step = duration of complete step cycle, Max = maximum value, Min = minimum value, rel_stan = percentage of stance phase in the total step cycle, SD = standard deviation of the mean; T-C = trot on concrete; T-R = trot on rubber mats.
